# Outcomes of Mohs Micrographic Surgery Compared to Wide Local Excision for Malignant Melanoma and Melanoma In Situ

**DOI:** 10.7759/cureus.107415

**Published:** 2026-04-20

**Authors:** Dora R Goldstein, Ryan S Koch, Radwa Emam, Kevin T Nguyen, Justin Raman, Floyd A Pirtle, Leor Daniel Goldstein, Michelle Tarbox, Kritin K Verma, Alba Posligua

**Affiliations:** 1 School of Medicine, Texas Tech University Health Sciences Center El Paso Paul L. Foster School of Medicine, El Paso, USA; 2 Department of Internal Medicine, Baylor Scott & White Medical Center - Temple, Temple, USA; 3 College of Medicine, Texas A&M College of Medicine, Bryan, USA; 4 School of Medicine, Texas Tech University Health Sciences Center, Lubbock, USA; 5 Department of Dermatology, Texas Tech University Health Sciences Center, Lubbock, USA; 6 Department of Public Health, The University of Texas at Austin, Austin, USA

**Keywords:** facial skin cancer, melanoma, mohs micrographic surgery, surgical outcomes, wide local excision

## Abstract

Mohs micrographic surgery (MMS), traditionally used for non-melanoma skin cancers, has been used for some cases of melanoma. Using the TriNetX (TriNetX, LLC, Cambridge, MA, USA) database, two analyses were performed. The first compared wide local excision (WLE) and MMS for the management of malignant melanoma (MM) of the face, scalp, and neck. The second analysis compared WLE and melanoma in situ (MIS) of the same anatomical areas. WLE was associated with a significantly higher incidence of secondary and unspecified malignant neoplasms of the lymph nodes of the head and neck and secondary malignant neoplasm of the bone, lung, and brain in the MM analysis. In the MIS analysis, WLE was found to have higher rates of secondary malignant neoplasms of the head and neck, bone, lung, and brain. The post-operative complication rates were similar between the MMS and WLE groups in both analyses.

## Introduction

As the incidence of melanoma continues to rise yearly, the need for optimized surgical treatments that preserve tissue, especially malignancies located on sensitive areas such as the head, genitals, and feet, becomes even more important [1]. Mohs micrographic surgery (MMS), traditionally used for non-melanoma skin cancers, has been used for cases of melanoma in situ (MIS), lentigo maligna, and thin lesions [1]. Mohs surgeons can achieve histologically clear margins with the use of immunohistochemical stains such as MART-1, SOX10, and human melanoma black-45 [1]. However, wide local excision (WLE) remains the standard surgical treatment for melanoma for all stages [2]. This study aims to compare postoperative complication rates of MMS vs. WLE, assess relative risks of secondary malignant neoplasms after each procedure, and evaluate whether MMS is comparable or superior in oncologic outcomes for malignant melanoma (MM) and MIS of the head and neck.

## Materials and methods

Using the TriNetX Research Network (TriNetX, LLC, Cambridge, MA, USA), two retrospective cohort analyses were conducted to compare outcomes between patients undergoing WLE and MMS for melanoma of the face, scalp, and neck. The first analysis included individuals diagnosed with MM, and the second focused on MIS of the same anatomical regions. 

Cohorts were identified using ICD-10 diagnostic and procedural codes [3], and patients were stratified based on whether they received WLE (Codes: 11640-11646) or MMS (Codes: 17311-17315) as their primary treatment. Subjects with outcomes prior to the index event, including baseline metastatic disease and same-day diagnoses, were excluded. Propensity score matching (1:1 greedy, nearest-neighbor matching with a caliper of 0.1 pooled standard deviations) was performed to generate demographically balanced cohorts for each analysis, controlling for age, sex, race, and ethnicity. This yielded matched samples of 11,032 patients per treatment group in the MM analysis and 12,977 patients per treatment group in the MIS analysis. Cohorts with MM were identified using ICD-10 codes C43.0, C43.1, C43.2, C43.3, and C43.5 while those with MIS were identified using ICD-10 codes D03.0, D03.1, D03.2, D03.3, and D03.4. Post-treatment outcomes were assessed using diagnostic codes recorded after the index surgical intervention, ensuring exclusion of pre-existing comorbidities. Risk ratios (RR) and 95% confidence intervals (CI) were calculated using Wald’s method, and statistical significance was defined at p < 0.05 using TriNetX.

A primary methodological limitation of this study is the undefined follow-up interval inherent to TriNetX, as diagnoses may be captured at varying time points after surgery. Consequently, while relative risks for secondary malignant neoplasms can be estimated, recurrence rates, disease-specific mortality, and timing of events cannot be reliably assessed. In the propensity score-matched cohorts of patients with MM and MIS treated with either WLE or MMS, demographic variables were comparable between treatment groups.

## Results

In the first analysis, a cohort of 11,032 patients with MM of the face, scalp, and neck treated with WLE and a cohort of 11,032 patients with the same malignancy treated with MMS were propensity-score matched (Table 1). Comorbidities were compared between the two cohorts. WLE was associated with a significantly higher incidence of secondary and unspecified malignant neoplasms of the lymph nodes of the head and neck (ICD-10 C77.0; RR 1.492, CI 95% 1.258-1.769, p< 0.0001) and secondary malignant neoplasm of the bone (ICD-10 C79.51; RR 1.259, CI 95% 1.071-1.48, p=0.0052), lung (ICD-10 C78.00; RR 1.441. CI 95% 1.248-1.664, p< 0.0001), and brain (ICD-10 C79.31; RR 1.589, CI 95% 1.342-1.881, p< 0.0001). For secondary and unspecified malignant neoplasm of axilla and upper limb lymph nodes (ICD-10 C77.3), the absolute difference between groups was small (0.707% vs 0.625%; absolute difference ≈0.08%), consistent with the non-significant RR. In contrast, for secondary and unspecified malignant neoplasm of lymph nodes (unspecified) (ICD-10 C77.9), the absolute difference was more pronounced (5.23% vs 3.73%; absolute difference ≈1.5%), which contextualizes the statistically significant RR (1.43) (Table 1). 

**Table 1:** Propensity-matched demographics of WLE and MMS for malignant melanoma of the face, scalp, and neck †After propensity score matching *Statistically significant values between cases and controls, defined at p < 0.05 CI: confidence interval; MMS: Mohs micrographic surgery; RR: risk ratio; WLE: wide local excision

Parameter	WLE treatment for MM of the face, scalp, and neck	MMS for MM of the face, scalp, and neck	RR (95% CI)†	P value†
Total participants (n)	11,032	11,032	–	-
Average age at index, SD (years)	71.9 ± 11.5	71.9 ± 11.5	–	0.915
Male	8,167 (74.03%)	8,167 (74.03%)	–	1
Female	2,786 (25.254%)	2,788 (25.272%)	–	0.975
Ethnicity	–	–	–	–
Not Hispanic or Latino	8,691 (78.78%)	8,690 (78.771%)	–	0.987
Hispanic or Latino	69 (0.625%)	70 (0.635%)	–	0.932
Race	–	–	–	–
White	10,328 (93.619%)	10,300 (93.365%)	–	0.445
Black or African American	10 (0.091%)	13 (0.118%)	–	0.531
Asian	10 (0.091%)	10 (0.091%)	–	1
Native Hawaiian or Other Pacific Islander	0 (0%)	10 (0.091%)	–	0.002*
American Indian or Alaska Native	10 (0.091%)	13 (0.118%)	–	0.531
Comorbidity	–	–	–	–
Secondary and unspecified malignant neoplasm of lymph nodes of the head, face, and neck	318 (2.883%)	216 (1.958%)	1.492 (1.258–1.769)	< 0.0001*
Secondary and unspecified malignant neoplasm of the axilla and upper limb lymph nodes	78 (0.707%)	69 (0.625%)	1.134 (0.821–1.567)	0.4433
Secondary and unspecified malignant neoplasm of lymph nodes (unspecified)	577 (5.230%)	411 (3.726%)	1.426 (1.26–1.613)	< 0.0001*
Hemorrhage (ICD-10 T81.0)	183 (1.659%)	221 (2.003%)	0.825 (0.679–1.002)	0.0515
Infection following a procedure (ICD-10 T81.4XXA)	207 (1.876%)	216 (1.958%)	0.953 (0.789–1.151)	0.6155
Facial nerve disorders (ICD-10 G51.9)	86 (0.780%)	97 (0.879%)	0.882 (0.661–1.178)	0.3963
Bell's palsy (ICD-10 G51.0)	64 (0.580%)	67 (0.607%)	0.952 (0.677–1.34)	0.7777
Disorders of the trigeminal nerve (ICD-10 G50.9)	45 (0.408%)	50 (0.453%)	0.9 (0.602–1.344)	0.6053
Secondary malignant neoplasm of bone	323 (2.928%)	256 (2.321%)	1.259 (1.071–1.48)	0.0052*
Secondary malignant neoplasm of the lung	439 (3.979%)	303 (2.747%)	1.441 (1.248–1.664)	< 0.0001*
Secondary malignant neoplasm of the brain	341 (3.091%)	214 (1.940%)	1.589 (1.342–1.881)	< 0.0001*
Malignant melanoma of lip	45 (0.408%)	30 (0.272%)	1.501 (0.946–2.38)	0.0823
Malignant melanoma of eyelid, including canthus	63 (0.571%)	59 (0.535%)	1.056 (0.741–1.505)	0.7626
Malignant melanoma of ear and external auricular canal	140 (1.269%)	114 (1.033%)	1.23 (0.962–1.572)	0.0983
Malignant melanoma of other and unspecified parts of face	322 (2.919%)	250 (2.266%)	1.124 (0.957–1.32)	0.1535
Malignant melanoma of scalp and neck	387 (3.508%)	262 (2.375%)	1.68 (1.442–1.959)	< 0.0001*
Malignant melanoma of trunk	336 (3.046%)	287 (2.602%)	1.177 (1.008–1.374)	0.0393*
Malignant melanoma of upper limb, including shoulder	259 (2.348%)	248 (2.248%)	1.054 (0.887–1.251)	0.5514
Melanoma in situ of lip	35 (0.317%)	29 (0.263%)	1.205 (0.737–1.969)	0.4568
Melanoma in situ of eyelid, including canthus	61 (0.553%)	44 (0.399%)	1.374 (0.933–2.023)	0.106
Melanoma in situ of ear and external auricular canal	155 (1.405%)	133 (1.206%)	1.144 (0.909–1.44)	0.2505
Melanoma in situ of other and unspecified parts of face	439 (3.979%)	432 (3.916%)	0.883 (0.777–1.004)	0.0582
Melanoma in situ of scalp and neck	426 (3.861%)	328 (2.973%)	1.318 (1.144–1.517)	0.0001*
Melanoma in situ of trunk	252 (2.284%)	247 (2.239%)	1.014 (0.852–1.206)	0.8771
Melanoma in situ of upper limb, including shoulder	225 (2.040%)	244 (2.212%)	0.922 (0.771–1.103)	0.3727
Melanoma in situ	597 (5.412%)	554 (5.022%)	0.829 (0.746–0.921)	0.0005*

Similarly, a cohort of 12,977 patients with MIS of the face, scalp, and neck treated with WLE and a cohort of 12,977 patients with the same malignancy treated with MMS were propensity-score matched (Table 2). WLE was found to have higher rates of secondary malignant neoplasms of the head and neck (RR 1.63, CI 95% 1.337-1.987, p<0.0001), bone (RR 1.241, CI 95% 1.042-1.478, p=0.0152), lung (RR 1.361, CI 95% 1.157-1.601, p=0.0002), and brain (RR 1.65, CI 95% 1.361-2.002, p<0.0001). In addition, patients treated with WLE had a significantly higher risk ratio of MIS and MM on the ear, face, scalp/neck, and trunk.

**Table 2:** Propensity-matched demographics of WLE and MMS for melanoma in-situ of the face, scalp, and neck †After propensity score matching *Statistically significant values between cases and controls, defined at p < 0.05 CI: confidence interval; MMS, Mohs micrographic surgery; RR: risk ratio; WLE: wide local excision

Parameter	WLE for MIS of the face, scalp, and neck	MMS for MIS of the face, scalp, and neck	RR (95% CI)†	P value†
Total participants (n)	12,977	12,977	–	–
Average age at index, SD (years)	71.8 ± 11.2	71.8 ± 11.2	–	0.907
Male	9,510 (73.284%)	9,522 (73.376%)	–	0.866
Female	3,365 (25.93%)	3,355 (25.853%)	–	0.887
Ethnicity	–	–	–	–
Not Hispanic or Latino	10,239 (78.901%)	10,242 (78.924%)	–	0.964
Hispanic or Latino	67 (0.516%)	73 (0.563%)	–	0.611
Race	–	–	–	–
White	12,153 (93.65%)	12,127 (93.45%)	–	0.511
Black or African American	10 (0.077%)	10 (0.077%)	–	1
Asian	10 (0.077%)	10 (0.077%)	–	1
Native Hawaiian or Other Pacific Islander	0 (0%)	10 (0.077%)	–	0.002*
American Indian or Alaska Native	10 (0.077%)	14 (0.108%)	–	0.414
Comorbidity	–	–	–	–
Secondary and unspecified malignant neoplasm of lymph nodes of the head, face, and neck	252 (1.942%)	156 (1.202%)	1.63 (1.337–1.987)	< 0.0001*
Secondary and unspecified malignant neoplasm of the axilla and upper limb lymph nodes	76 (0.586%)	59 (0.455%)	1.292 (0.92–1.814)	0.1376
Secondary and unspecified malignant neoplasm of lymph nodes (unspecified)	454 (3.498%)	324 (2.497%)	1.42 (1.234–1.633)	< 0.0001*
Hemorrhage	201 (1.549%)	211 (1.626%)	0.95 (0.784–1.151)	0.6009
Infection following a procedure	238 (1.834%)	208 (1.603%)	1.142 (0.95–1.373)	0.1581
Facial nerve disorders	103 (0.794%)	92 (0.709%)	1.118 (0.845–1.479)	0.4361
Bell's palsy	81 (0.624%)	60 (0.462%)	1.349 (0.967–1.882)	0.0771
Disorders of the trigeminal nerve	49 (0.378%)	44 (0.339%)	1.113 (0.741–1.671)	0.6048
Secondary malignant neoplasm of bone	277 (2.135%)	223 (1.718%)	1.241 (1.042–1.478)	0.0152*
Secondary malignant neoplasm of the lung	337 (2.597%)	247 (1.903%)	1.361 (1.157–1.601)	0.0002*
Secondary malignant neoplasm of the brain	269 (2.073%)	163 (1.256%)	1.65 (1.361–2.002)	< 0.0001*
Malignant melanoma of the lip	43 (0.331%)	32 (0.247%)	1.346 (0.852–2.125)	0.2012
Malignant melanoma of the eyelid, including the canthus	89 (0.686%)	68 (0.524%)	1.306 (0.953–1.789)	0.0953
Malignant melanoma of the ear and external auricular canal	214 (1.649%)	141 (1.087%)	1.521 (1.232–1.879)	< 0.0001*
Malignant melanoma of other and unspecified parts of the face	746 (5.749%)	533 (4.107%)	1.41 (1.267–1.569)	< 0.0001*
Malignant melanoma of the scalp and neck	523 (4.030%)	358 (2.759%)	1.594 (1.397–1.818)	< 0.0001*
Malignant melanoma of the skin	797 (6.142%)	703 (5.417%)	1.438 (1.312–1.577)	< 0.0001*
Malignant melanoma of the trunk	367 (2.828%)	272 (2.096%)	1.364 (1.168–1.592)	< 0.0001*
Malignant melanoma of the upper limb, including the shoulder	287 (2.212%)	248 (1.911%)	1.17 (0.989–1.384)	0.0665
Melanoma in situ of the lip	38 (0.293%)	32 (0.247%)	1.186 (0.742–1.897)	0.4751
Melanoma in situ of the eyelid, including the canthus	72 (0.555%)	55 (0.424%)	1.303 (0.918–1.85)	0.1367
Melanoma in situ of the ear and external auricular canal	140 (1.079%)	105 (0.809%)	1.326 (1.031–1.705)	0.0276*
Melanoma in situ of other and unspecified parts of the face	309 (2.381%)	229 (1.765%)	1.183 (1.001–1.397)	0.0476*
Melanoma in situ of the scalp and neck	293 (2.258%)	180 (1.387%)	1.773 (1.476–2.13)	< 0.0001*
Melanoma in situ of the trunk	339 (2.612%)	276 (2.127%)	1.239 (1.059–1.449)	0.0073*
Melanoma in situ of upper limb, including shoulder	298 (2.296%)	262 (2.019%)	1.148 (0.974–1.353)	0.0992

## Discussion

Causality cannot be inferred from the data in both analyses due to the retrospective nature. These findings suggest that a possible benefit associated with treatment with MMS is based on ICD-10 coding and may not reflect confirmed metastases and could be due to selection bias; however, a benefit with MMS was seen in previous research [4-6]. Taylor et al. (2024) reported that MMS presented with superior disease-specific survival compared to WLE for head and neck cutaneous melanomas in a large U.S. cohort, while Cheraghlou et al. (2019) found enhanced overall survival in early-stage invasive melanoma treated with MMS versus wide excision [5,6]. Similarly, Balado-Simó et al. (2025) emphasized that MMS allows for maximal tissue conservation with precise histologic margin control, potentially improving both oncologic and functional outcomes [4].

In contrast, some studies have reported comparable recurrence and complication rates between MMS and WLE, suggesting that the benefit of MMS may depend on tumor location, depth, and histologic subtype. The post-operative complication rates were similar between the MMS and WLE groups in both analyses performed, indicating that the primary advantage of MMS may correspond with achieving local control and minimizing metastatic risk rather than in reducing surgical morbidity.

This study was limited by the diagnoses and ICD-10 coding provided in patient health records, and the ICD-10 codes for secondary malignant neoplasms are miscoded rather than actual metastasis. Additionally, due to limitations with TriNetX, the risk of local recurrence or disease-specific deaths could not be analyzed, and key melanoma prognostic factors such as tumor depth, ulceration, stage, and subtype could not be included in the propensity score matching. While prospective, randomized studies would be needed to determine causality, the patterns identified in this analysis should be interpreted as correlations rather than evidence of treatment superiority.

## Conclusions

In this retrospective analysis using the TriNetX database, Mohs micrographic surgery (MMS) demonstrated comparable postoperative complication rates to wide local excision (WLE) while showing a lower relative risk of coded secondary malignancies in patients with malignant melanoma and melanoma in situ on the face, scalp, and neck. These findings infer that MMS may offer oncologic outcomes comparable to or potentially preferred to WLE, specifically in anatomically sensitive areas where tissue preservation is critical. While causality cannot be established due to the retrospective nature of the data and limitations in coding accuracy, the consistency of these results with prior studies underscores the need for prospective, randomized investigations to further evaluate the role of MMS in melanoma management. Overall, these results emphasize the potential of MMS as a tissue-sparing alternative for select melanomas without compromising oncologic safety. Incorporating MMS into treatment algorithms for head and neck melanomas may improve both functional and cosmetic outcomes while maintaining comparable survival benefits.
